# Combined Solid Organ Transplant and Transfusion-Associated Graft-Versus-Host Disease in a Lung Transplant Recipient: An Uncertain Culprit With Lethal Complications

**DOI:** 10.1155/crit/3034200

**Published:** 2024-12-21

**Authors:** Devika Sindu, Brian J. Franz, Ian Scott, Hashem Ayyad, Kristina Gaines, Kendra McAnally, Sofya Tokman

**Affiliations:** ^1^Norton Thoracic Institute, St. Joseph's Hospital and Medical Center, Phoenix, Arizona, USA; ^2^Histocompatibility Laboratory, Vitalant, Phoenix, Arizona, USA; ^3^Anatomic Pathology, St. Joseph's Hospital and Medical Center, Phoenix, Arizona, USA; ^4^Creighton University School of Medicine, Phoenix Health Sciences Campus, Phoenix, Arizona, USA

**Keywords:** bone marrow, chimerism, graft-versus-host disease, lung transplant, transfusion

## Abstract

Although graft-versus-host disease (GVHD) is a common complication of hematopoietic stem cell transplantation, it is rare after solid organ transplantation (SOT) or blood transfusion. We present a rare case of SOT-derived and/or transfusion-associated graft-versus-host disease (TA-GVHD) in a 66-year-old man with interstitial lung disease who underwent bilateral lung transplantation (LT) from a 12-year-old female donor and required three units of packed red blood cells intraoperatively. He presented with signs and symptoms consistent with GVHD, and a bone marrow biopsy revealed an XX karyotype. He died 3 months after bilateral LT, and postmortem chimerism testing using next-generation sequencing identified three sources of DNA within his bone marrow, including the recipient, the lung donor, and a third donor, thereby suggesting the presence of solid organ transplant graft-versus-host disease (SOT-GVHD), TA-GVHD, or a combination of both.

## 1. Introduction

Graft-versus-host disease (GVHD) is a rare complication of blood transfusion and solid organ transplantation (SOT) [1]. Patients can present with an erythematous, maculopapular rash, diarrhea, fever, hepatomegaly, transaminitis, marrow aplasia, and pancytopenia [2]. Diagnosis and treatment may be delayed as cases of GVHD are rare outside of hematopoietic stem cell transplantation (HSCT), and symptoms can be attributable to other posttransplant complications [3, 4]. Irradiation of blood products prevents transfusion-associated graft-versus-host disease (TA-GVHD) but also reduces the shelf life of packed red blood cells (pRBCs), delays the release of blood components, and increases cost [5, 6]. There are no known preventative strategies for solid organ transplant graft-versus-host disease (SOT-GVHD), and there is no consensus for diagnosis or treatment. We present a case of likely combined SOT-GVHD and TA-GVHD in a lung transplant recipient (LTR), as confirmed by postmortem chimerism testing.

## 2. Case Presentation

**Figure 1:**
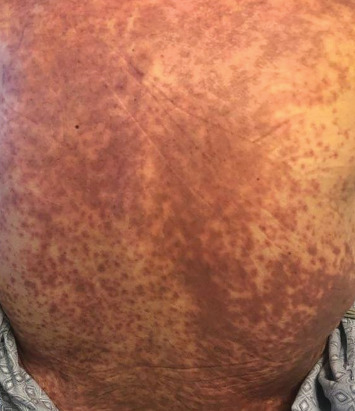
Widespread erythematous maculopapular rash seen 42 days after lung transplant. The rash initially developed on his upper back, 33 days after lung transplant, and spread to his entire back, chest, abdomen, and neck but was not associated with any mucosal involvement.

**Figure 2:**
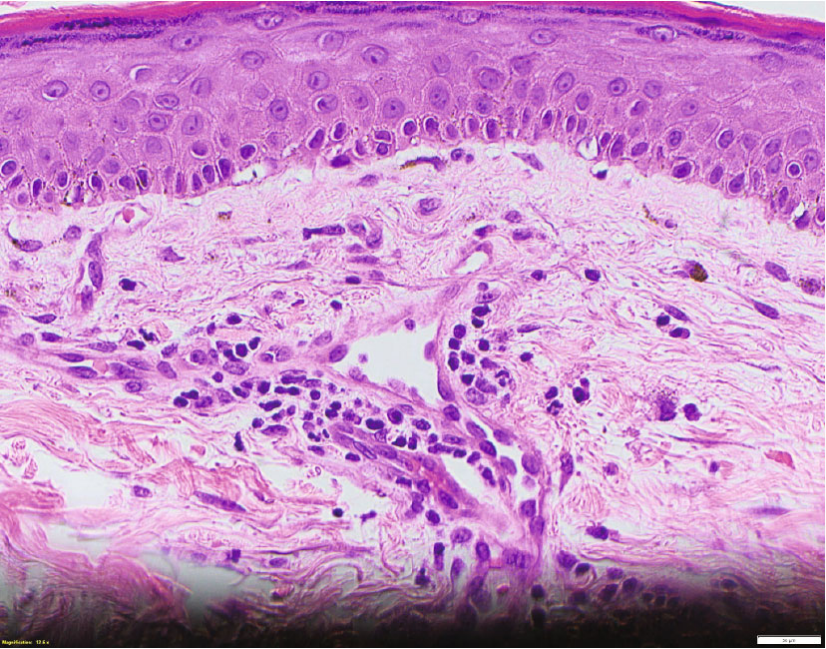
An anterior abdominal wall skin punch biopsy shows mild superficial perivascular dermatitis (hematoxylin and eosin stain; original magnification ×40). Sections reveal primarily lymphocytes, plasma cells, monocytes, and focal red blood cell extravasation with scattered dermal melanophages (iron stain negative).

A 66-year-old man with pulmonary fibrosis received a bilateral lung transplantation (LT) from a 12-year-old female donor. His pre-LT infectious workup revealed the following relevant antibody serologies: varicella zoster virus (VZV) IgG (+), Epstein–Barr virus (EBV) IgG (−), cytomegalovirus (CMV) IgG (−), herpes simplex virus (HSV) 1 and 2 IgG (+), rubella (+), and rubeola IgG (+); human herpesvirus (HHV) 6 or 8 serologies are not routinely checked at our institution. His crossmatch results were negative, and he showed no pre-LT allosensitization. At the time of transplant, he was transfused three units of pRBCs, one from a female donor and two from a male donor. The pRBC units were leukoreduced but not irradiated. Serologically, the recipient (R) and the donor (D) were both CMV negative (D−/R−) but EBV mismatched (D+/R−). His post-LT course was complicated by ileus, dysphagia, and a pulmonary embolism. He was discharged from the hospital a month after LT on a standard three-drug immunosuppressive regimen (prednisone, tacrolimus, and mycophenolate mofetil) and opportunistic infection prophylaxis (valganciclovir, sulfamethoxazole–trimethoprim, and itraconazole). He was readmitted 4 days after hospital discharge with fever, transaminitis, and a nonpruritic, erythematous, maculopapular rash (Figure 1). Drug reaction with eosinophilia and systemic symptoms (DRESS) syndrome was suspected, and the culprit medications were stopped. However, his clinical status worsened, and he was transferred to the intensive care unit for delirium, respiratory failure, and acute kidney injury. His infectious workup revealed EBV viremia (5.8 log IU/mL), without posttransplant lymphoproliferative disorder, and HHV6 type B viremia (5.4 log/mL or 253,000 copies/mL); however, HSV, VZV, and parvovirus PCR testing results remained negative. He subsequently developed rapidly progressive pancytopenia, raising suspicion for drug-induced bone marrow failure versus hemophagocytic lymphohistiocytosis. He underwent two bone marrow biopsies (41 and 47 days after transplant) that showed severe pancytopenia, no hemophagocytic lymphohistiocytosis, and an XX karyotype. His subsequent skin biopsy was consistent with superficial perivascular dermatitis (Figure 2) or a drug reaction and a mixed XX/XY karyotype, with no evidence of viral inclusions. Mycophenolate mofetil was reduced and subsequently stopped, and he was treated with high-dose corticosteroids. However, bone marrow failure progressed, and he died from septic shock 3 months after LT.

Postmortem double-donor chimerism testing was performed using next-generation sequencing technology (targeting 202 biallelic single nucleotide polymorphisms across 22 autosomes) using pre-LT blood samples from the donor and the recipient and post-LT formalin-fixed paraffin-embedded (FFPE) bone marrow samples collected from the recipient. The deoxyribonucleic acid (DNA) from the bone marrow samples was isolated using the Quick-DNA™ FFPE Kit (Zymo Research Corporation). Chimerism testing of the first bone marrow biopsy detected an average of 90.7% recipient-derived DNA, 8.8% lung-donor-derived DNA, and 0.5% of DNA derived from a third source; the same testing of the second bone marrow biopsy detected 85.3% recipient-derived DNA, 9.4% lung-donor-derived DNA, and 5.3% of DNA from a third source. Although the identity of the third source of DNA is uncertain, we assume it is transfusion derived. Thus, the results of chimerism testing suggest the presence of SOT-GVHD and possible concurrent TA-GVHD.

## 3. Discussion

GVHD occurs due to the translocation of immunologically competent donor-derived lymphocytes from the transplanted organ or transfused blood product into the immunocompromised recipient who is unable to eliminate these cells [7, 8]. These lymphocytes become activated and injure recipient tissues, including the skin, liver, bone marrow, and gastrointestinal tract [9]. Among SOT recipients, small bowel recipients have the highest risk of GVHD, with an incidence of approximately 5%–10% [10]. In contrast, GVHD in LTRs is rare, with a literature search revealing only 11 reported cases in LTRs and six cases in combined heart-lung transplant recipients [11].

Irradiation remains the most reliable method of inactivating lymphocytes in transfused blood components, thereby eliminating the risk of TA-GVHD [12]. Current guidelines recommend the use of irradiated blood components in patients receiving alemtuzumab and purine analogues for hematologic disorders or allogeneic or autologous HSCT; however, the use of irradiated blood components in SOT recipients is not universally required in the United States, Australia, or the United Kingdom [5, 6, 13]. Leukoreduction, which may help lower but not eliminate the risk of TA-GVHD, has become the standard of care at many facilities. Our patient received leukoreduced but not irradiated blood, per our institution's policy as he had no history of HSCT. A report by Pavenski et al. [14] describes a young female LTR who succumbed to SOT-GVHD and concomitant macrochimerism from transfusions. The authors of this case also highlight the vital need for further studies to determine whether superimposed TA-GVHD can cause further deterioration in the patient's clinical status, which may warrant the use of irradiated blood products in select SOT recipients.

Unlike TA-GVHD, the risk factors for SOT-GVHD remain unclear; however, a high degree of iatrogenic or innate immunosuppression likely plays a role. A recent report by Brestoff et al. [15] described the detection of a loss-of-function mutation in the telomerase reverse transcriptase (*TERT*) gene in a patient with familial pulmonary fibrosis who died from acute SOT-GVHD after LT. Abnormally low telomerase activity is associated with impaired immunosurveillance and defective cell-mediated immunity, and the report by Brestoff et al. suggests that loss-of-function mutations in *TERT* may reduce a recipients ability to effectively eliminate donor immune cells in lung grafts [15, 16]. Our patient had underlying pulmonary fibrosis and may have had telomerase dysfunction; however, he was not tested for telomerase mutations or short telomeres during the pre-LT evaluation as he denied a consistent family history.

The diagnosis of GVHD is challenging, and previous reports have shown that chimerism testing can be helpful as it detects donor cells in blood and tissue [17–21]. However, chimerism testing should likely be reserved for patients with a high likelihood of having GVHD, as the clinical utility of the test may be limited by the possibility of asymptomatic donor chimerism [22]. Similarly, a higher proportion of chimerism may not translate to a worse clinical outcome [14]. Our patient's clinical presentation in conjunction with chimerism testing that detected recipient-derived DNA, lung-donor-derived DNA, and DNA derived from a third source suggests that he was likely suffering from SOT-GVHD or TA-GVHD, or a combination of both. Both SOT-GVHD and TA-GVHD carry a poor prognosis with a high mortality rate despite aggressive treatment, thereby underscoring the need for early diagnosis and treatment.

Prompt diagnosis of SOT-GVHD and TA-GVHD is hindered by multiorgan system involvement and overlapping presentation with more common clinical syndromes, such as adverse drug effects, including more severe medication-related complications like DRESS, and systemic viral infections. Pursuing all of these diagnoses in parallel rather than in sequence is prudent and involves cessation of potential culprit medications, close collaboration with infectious disease and hematology consultants, tissue sampling of effected organ systems, and chimerism testing.

## Data Availability

Data sharing is not applicable to this article as no new data were created or analyzed in this study.

## Nomenclature

DNA: deoxyribonucleic acid
FFPE: formalin-fixed paraffin-embedded
GVHD: graft-versus-host disease
HSCT: hematopoietic stem cell transplantation
LT: lung transplantation
LTR: lung transplant recipient
pRBCs: packed red blood cells
SOT: solid organ transplantation
TA: transfusion-associated
*TERT*: telomerase reverse transcriptase (gene)
